# Cervical amyloidoma of transthyretin type: a case report and review of literature

**DOI:** 10.1186/s12877-022-03422-8

**Published:** 2022-09-15

**Authors:** Matthew H. MacLennan, André le Roux

**Affiliations:** 1 Department of Medicine, Dalhousie Medicine New Brunswick, 100 Tucker Park Road, Saint John, New Brunswick E2K 5E2 Canada; 2 Division of Neurosurgery, Department of Surgery, Horizon Health Network, Saint John, New Brunswick Canada; 3 Canada East Spine Centre, Saint John, New Brunswick Canada

**Keywords:** Amyloidoma, Transthyretin, Cervical spine, Spinal decompression and fusion, Case report

## Abstract

**Background:**

Amyloidoma is a rare clinical entity characterized by the focal aggregation of amyloid protein within the body, void of systemic involvement. To our knowledge, there have only been 26 reports of cervical amyloidoma to date. Amyloid light chain and beta-2-microglobulin are the most common types, with only three previous reports of transthyretin (ATTR) Amyloidoma.

**Case presentation:**

We report a case of a 71-year-old male who presented with worsening strength and coordination of his upper extremities, right upper-leg pain, unsteady gait, and a reduced range of motion of his neck in all planes. Magnetic resonance imaging revealed a solitary mass compressing the spinal cord at C1-C2. Treatment consisted of cervical decompression and stabilization. Pathological examination confirmed solitary amyloid deposition of ATTR. Postoperative neurological assessment revealed improved balance, gait, hand function, and grip strength. Investigational imaging was ordered 8 months postoperatively revealing no evidence of systemic involvement, confirming the diagnosis of cervical ATTR amyloidoma. A discussion is provided surrounding the published literature of ATTR amyloidoma with description of the typical presentation, management, and outcomes of this rare pathology.

**Conclusion:**

Previous cases and studies indicate clinical signs such as ligamentum of flavum hypertrophy and carpal tunnel syndrome may precede focal ATTR spinal disposition. Outcomes for amyloidoma are generally favourable, as tumour resection prevents irreversible deficits. Patients have a low rate of recurrence with an overall excellent prognosis following resection and stabilization.

## Background

Amyloidosis is a disease caused by the aggregation of amyloid which are insoluble fibrils with a cross beta-pleated sheet structure made of an assembly of misfolded and normally soluble proteins [1]. Aggregates deposit in the extracellular spaces of organs and tissues in either a systemic or local fashion [2, 3]. The origin of the term dates to 1854 where it was introduced by Rudolph Virchow when describing a tissue with an abnormal macroscopic appearance. Originally thought to be related to cellulose or starch, later experiments revealed its proteinaceous nature [3].

Amyloidoma is characterized by focal depositions that do not feature systemic involvement. Amyloidomas found in the spine are a rare clinical entity. The first report of cervical amyloidoma was published in 1988 by Dickman et al. and to our knowledge, there have only been 26 reports of amyloidoma identified in the cervical spine [4–29]. We present a case of a 71-year-old patient with transthyretin (ATTR) type cervical amyloidoma and provide a review of literature on amyloidoma involving the cervical spine.

## Case presentation

A 71-year-old man presented to his family physician with upper right leg pain. His symptoms progressed with deterioration in his balance and decreased strength and coordination in his upper limbs. Functionally, the patient remarked being clumsy at home, dropping objects on a regular basis, unable to do buttons and/or zippers, and excluded several activities of daily living due to these limitations. The patient had no previous spinal trauma and an unremarkable family history. His previous surgical history included a bilateral hip replacement, tonsillectomy, and carpal tunnel release. A referral was then made to neurosurgery for further management.

Neurological examination revealed an unsteady gait with the inability to heel-to-toe walk and a positive Romberg’s sign. He was unable to sit comfortably and exhibited a reduced range of motion of the neck in all planes. Examination of his upper limbs revealed deficits that were particular to his right side. This included atrophy in the first web space, decreased grip strength (4−/5), poor finger function and reduced sensation. Left arm and hand involvement was present but to a lesser degree. Lower limb involvement included decreased sensation in both feet and in the right L3 dermatome. Reflexes were brisk.

Due to the neurological findings, computed tomography (CT), and nerve conduction study (NCS) were obtained. NCS revealed no abnormal findings while the CT scan showed a mass (28x15x39mm) that compressed the cervical spinal cord at C2.

**Fig. 1:**
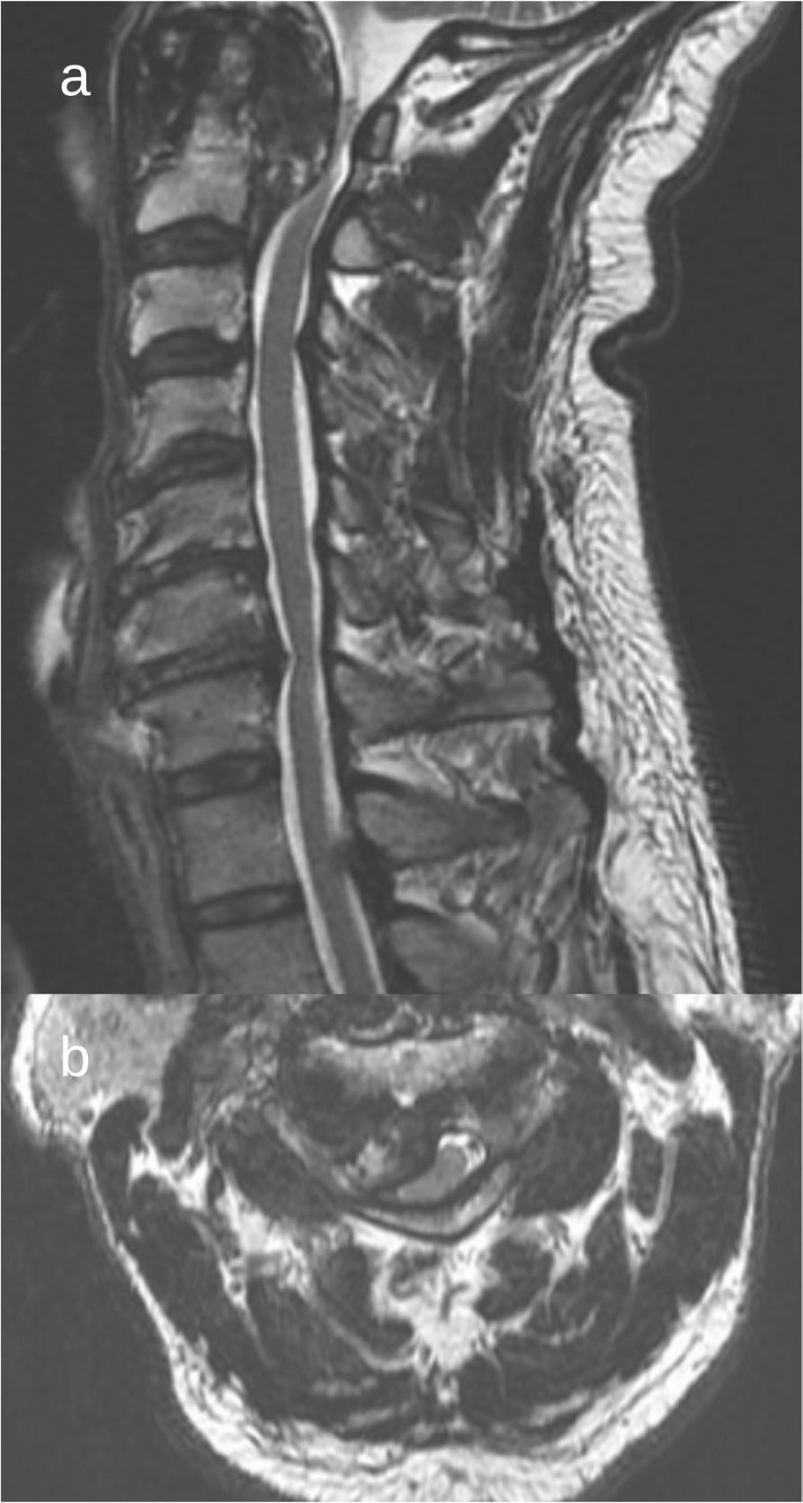
Preoperative MRI T2-weighted imaging: sagittal (**a**) and axial (**b**) views suggesting a congenitally narrow cervical spinal canal and hypointense mass causing significant spinal cord compression at C1 and C2

Magnetic resonance imaging (MRI) was obtained revealing a congenitally narrow cervical spinal canal and significant compression of the spinal cord at C1 and C2 (Fig. 1). Some canal stenosis, in the thoracic and lumbar regions, was present. This was secondary to degenerative changes and ligamentous hypertrophy.

A diagnosis of cervical myelopathy secondary to a C2 mass was made. Informed consent for surgery was obtained. Surgical management consisted of decompression of the cervical spinal cord with a posterior cervical fusion of C1-C3. Tissue biopsies were obtained and sent to pathology for investigation.

**Fig. 2:**
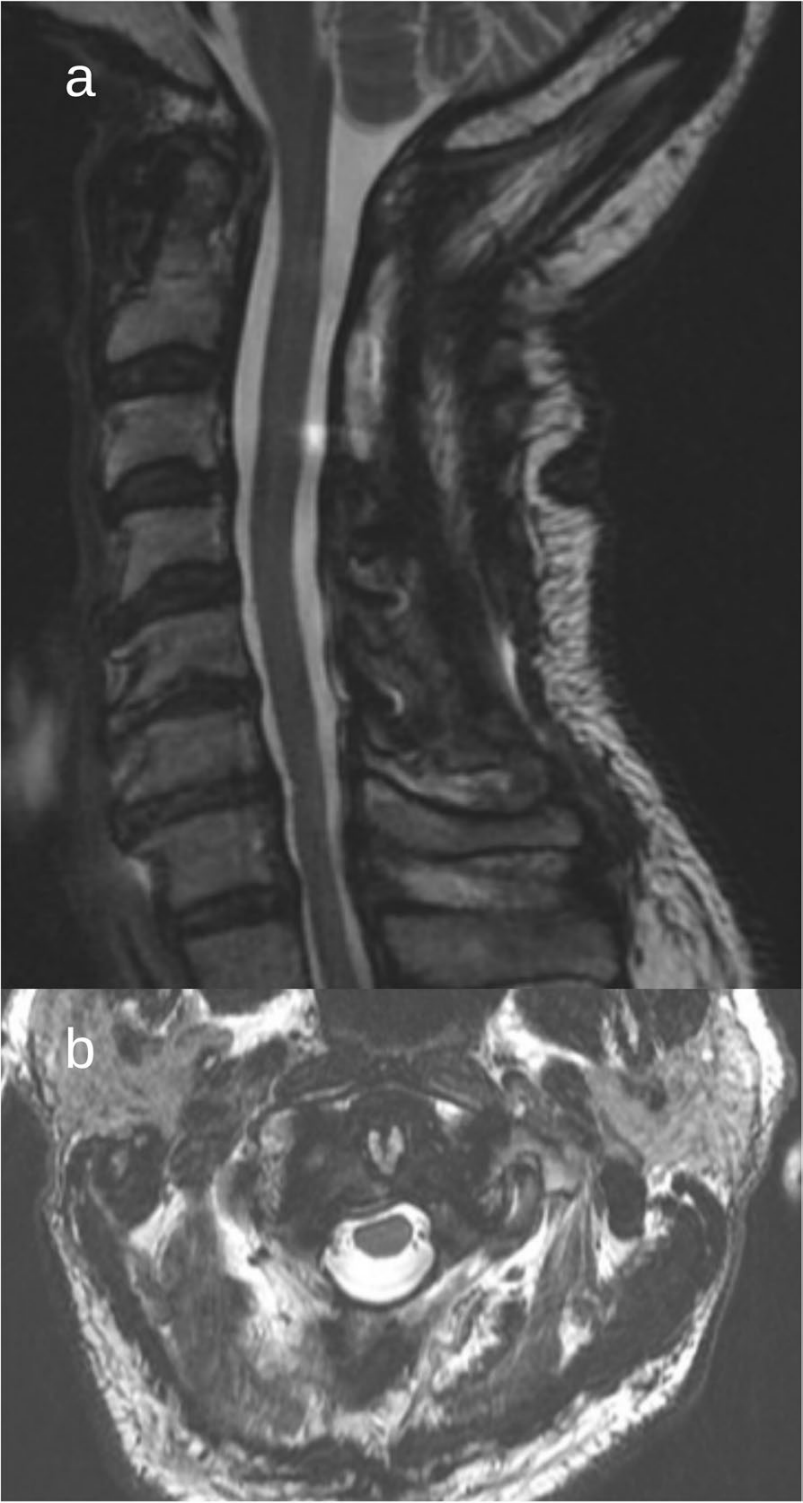
Postoperative MRI T2-weighted imaging: sagittal (**a**) and axial (**b**) views suggested substantial spinal cord decompression at C1 and C2

CT imaging was performed in the early postoperative period which demonstrated no bony or hardware complications. MRI imaging was done 6 months postoperatively (Fig. 2) which illustrated the decompression of the cervical spinal cord. Follow-up clinical examination at that stage found increased grip strength, mobility, and balance with an overall improvement as compared to pre-operative symptomology. He was referred to physiotherapy to aid with rehabilitation.

Further radiological investigations were obtained to verify that the mass was not secondary to systemic amyloidosis. Results were negative, leading to the confirmation of primary solitary amyloidosis treated with uncomplicated cervical spine surgery.

Furthermore, the patient has been referred to Cardiology and completed a MIBI scan with no evidence of cardiac infiltration. Additional cardiac studies and genetic assessment are planned but have not yet been completed at the time of this report; however, it is suspected the ATTR subtype is non-genetic in nature. The patient is receiving further work-up regarding his lumbar spinal stenosis as it requires treatment.

## Pathological findings

Histological examination of the biopsy revealed positive Congo red stained tissue, proof of amyloid deposition [30]. Congo red positive areas were blocked in paraffin and sent for proteomic analysis by mass spectrometry which indicated amyloid deposition of ATTR type.

## Discussion and conclusions

Classification of amyloidosis has evolved over the years. Currently, nomenclature consists of beginning with protein A, the amyloid fibril protein, followed by the abbreviated name of the precursor protein. Presently, there are 40 known human amyloid fibril proteins, most of which are exceedingly rare [3]. Of the known proteins, commonly reported amyloidoma types include immunoglobin light chain (AL), serum amyloid A (AA), and β2-microglobulin (Aβ2M); less commonly is the transthyretin type [21, 28]. Most often, reported cases are attributed to either AL or﻿ Aβ2M types [2, 21].

AL is a disorder of plasma cells, where lambda or kappa light chains aggregate in focal dispositions and is associated with multiple myeloma patients [31]. AA is often referred to as secondary amyloidosis, as the disease is secondary to longstanding inflammation caused by diseases such as rheumatoid arthritis, ﻿chronic osteomyelitis, or Crohn’s Disease [30]. Aβ2M is caused by renal failure in patients with long-standing dialysis who have decreased clearance of the protein A beta-2-microglobulin. Transthyretin functions as a transport protein for thyroxin and retinol and is mainly produced in the liver, whereas small amounts are produced by the choroid plexus. ATTR is commonly categorized as wild-type (ATTRwt) and variant (ATTRv), otherwise defined as the hereditary subtype [32]. Additionally, a third subtype has been reported in patients who receive liver transplants from ATTRv donors, referred to as acquired ATTR amyloidosis [32]. ATTRwt is characterized by normal TTR protein aggregation, while ATTRv is tied to inherited autosomal dominant point mutations [30].

ATTRwt amyloidosis is classically associated with cardiomyopathy in the elderly, more commonly in male patients; however, features of carpal tunnel syndrome and spinal canal stenosis. ATTRv Amyloidosis has been studied within endemic and non-endemic populations which have revealed different clinical features both between endemic and non-endemic populations, as well as between endemic populations such as Sweden compared to Japan and Portugal [32]. Physical manifestations of ATTRv are broad and include neuropathy, cardiomyopathy, oculoleptomeningeal involvement, and potentially myopathy [2, 32, 33]. Acquired ATTR amyloidosis may develop after domino liver transplantation, as the donor’s functioning liver continues to produce ATTRv, and studies have shown the mean duration from transplantation to symptom onset is roughly 8 years [32]. It should be noted that the symptoms differ between the recipient and the donor’s original symptoms. Recipient symptomology tends to focus on sensory, not autonomic symptoms [32].

Despite the rare occurrence of solitary focal deposits of ATTR along the spinal canal, several studies have investigated the effects of transthyretin amyloidosis in other areas of the human body. ATTRwt disposition has been commonly identified within the ligamentum of flavum (LF) associated with spinal stenosis, the transverse carpal ligament associated with carpal tunnel syndrome (CTS), and endomyocardial tissues affiliated with heart failure [34–36]. More recently, studies have shown CTS commonly precedes ATTR amyloidosis [37, 38].

Our patient’s history was significant of carpal tunnel release and radiological assessment revealed compression in both lumbar and thoracic regions due to hypertrophy of the ligaments. To our knowledge, there have been only three previous cases with spinal involvement that have demonstrated a positive or slightly positive result for the transthyretin type [10, 19, 25]. Table 1 illustrates a comparison of the 26 cases identified in our literature review regarding amyloidoma involving the cervical spine.

**Table 1:** Summary of cervical spine amyloidoma cases within the literature

Reference	Age	Sex	Clinical Symptoms	Imaging	Treatment	Outcome	Histological Description	CTS Hx	Spinal Stenosis
**Dickman 1998** [4]	74	M	Upper cervical pain radiating to occiput and shoulders.	CT: C2 mass with bubbly-appearing cortical shell and complete central lucency.	Open biopsy and tumor resection via midline posterior approach. Second surgery involved fusion of C1-C3 with iliac bone graft.	Postoperatively exhibited no new neurological deficits. Patients died 3-months postoperative due to sudden myocardial infarction.	Demonstrated waxy-appearing Congo red-positive substance with green birefringence to polarized light.	Not reported.	Not reported.
**Mullins 1997** [5]	58	M	Chest discomfort in midsternal/epigastric region precipitated by coughing and valsava.	MRI T1: Abnormal enhancement of the C7 vertebral body and posterior elements.	C6-C7 laminectomy and corpectomies completed with iliac bone graft and C4-T2 fusion.	12-month follow up revealed stable spine construct and no evidence of recurrence.	Stains revealed Congo red stain for amyloid, and green birefringence under polarized light.	Not reported.	Not reported.
**Porchet 1998** [6]	73	M	6-year history of progressive numbness and spasticity in all limbs, predominantly the right side, dragging right leg, and neck pain.	1991 MRI: partially enhancing C1-C2 mass with odontoid erosion. 1995 MRI: enlargement of enhancing mass found from the clivus-C2.	1991: laminectomy from C1-C4. 1995: Trans-oral odontoidectomy with resection; C1-C2 transarticular screw fixation.	1991: Decreased right hand numbness, increase strength and coordination in all extremities, resuming to normal activity. 1995: At 7-months postop, able to ambulate half a mile, numbness resolved, and right lower limb strength returned to normal.	Positive Congo red stain with apple-green birefringence under polarized light. Immuno-histochemical staining was positive for ﻿β2M.	Bilateral carpal tunnel release in 1981, although this did not improve symptoms.	Not reported.
**Moonis 1999** [7]	79	M	Progressive dysphagia, weakness in his arms and legs, rapid weight loss, and neck pain.	MRI: Peri-odontoid 3 × 2 cm hypointense mass.	Anterior microdissection of the tumor and posterior fusion from occiput to C3.	Not reported.	Stained positive for amyloid using Congo red stain. β2M antibodies revealed intense staining.	Carpal tunnel release 2 years prior.	Not reported.
**Hwang 2000** [8]	45	F	Paraparesis, urinary incontinence, and 3-month long neck pain.	MRI: Inhomogeneous hypointense mass at C7 with partial collapse of the bony anatomy.	Decompressive corpectomy with anterior fusion from C6-T1.	After the operation, the patient’s paraparesis and urinary incontinence resolved completely. 3-year follow-up reported no specific symptoms.	Positive Congo red stain, with green birefringence to polarized light.	Not reported.	Not reported.
**Mulleman 2004** [9]	79	F	Acute cervical pain and spastic tetraparesia occurring after a fall.	MRI: Peripheral enhancing hypointense retro-odontoid mass with compression at C1-C2.	Transoral approach for removal of mass. Underwent C1-C2 transarticular screw fixation 3-weeks postop.	Improved sphincter tone and strength in all limbs. Improved neurological function post rehabilitation.	Slightly positive for prealbumin/ATTR subtype.	Yes.	Not reported.
**Shenoy 2004** [10]	58	M	Neck pain, progressive weakness of the limbs, and dysphagia for 2 months.	MRI T1: hypointense mass with bony destruction at C1-C2.	Intubated and given ventilatory support due to rapid decline in respiratory function.	Progressively worsened and developed bleeding diathesis and died.	Post-death transoral biopsy revealed amyloid deposits under Congo red stain with apple-green birefringence under polarized light.	Not reported.	Not reported.
**Belber 2004** [11]	72	M	Acute non-radicular left arm pain, followed by mild right arm and leg pain.	MRI: C1-C3 hypointense lesion.	Bilateral C2 decompression and partial C1 laminectomy.	Discharged home day 10 postop fully ambulatory. Died 3 months later during management of MM.	Exhibited typical apple green birefringence under polarized light on Congo Red stain.	Not reported.	Not reported.
**Samandouras 2006** [12]	75	F	Progressive lower limb stiffness over 16 years.	MRI T2: C2 hyperintense pannus with a cystic lesion. Mild erosion present on posterior cortical margin of C2.	C1-C2 laminectomy with excision of the cystic mass.	Not reported.	Positive for Congo red stain. Presumed to be AL, immune-histochemistry studies included AA and ATTR types.	Not reported.	Not reported.
**Iplikcioglu 2007** [13]	72	M	4-year history of progressive weakness and numbness in both upper limbs, and neck pain.	MRI: Soft tissue isointense mass at C6-C7 with vertebral body destruction.	Anterior approach for resection of the C6-C7 mass with acrylic vertebroplasty.	Uneventful postoperative course and quadriparesis was decreased.	Tissue stained with Congo red under polarized light revealed yellow-green birefringence with deposits of primary (AL) amyloid.	Not reported.	Not reported.
**Vignes 2007** [14]	50	F	Paresthesia in the hand and shoulder with progressive cervical pain.	MRI T1: hypointense C2-C3 lateral mass	C2-C3 laminectomy and tumor excision.	Improvement of neuralgia syndrome and neuropathic pain; however, required a cervical collar for 3 months.	Positive Congo red stain and birefringent under polarized light.﻿ Immuno- histochemistry revealed presence of β2M.	History of bilateral carpal tunnel operation with recurrent syndrome.	Not reported.
**Oruckaptan 2009** [15]	47	M	Headache	MRI T1: hyperintense C1-C2 lesion with hypointense center.	Complete resection of the lesion and concomitant acrylic cranioplasty.	Uneventful postoperative course.	Congo red and crystal violet dyes verified diagnosis of amyloidoma. Protein electrophoresis showed β2M.	Not reported.	Not reported.
**Farrell 2011** [16]	75	M	Right leg weakness and paresthesia in all four limbs followed by acute quadriparesis.	MRI: Paravertebral isointense soft tissue mass from C6-C7.	Radiotherapy, surgical debulking, Bortezomib, and Dexamethasone.	Cervical amyloidoma diagnosed in 1993, with symptoms returning in 2006. Final treatment in 2009 led to gradual improvement in leg power and normal arm power over 8-months.	Congo red staining demonstrated apple-green birefringence. Noted to express lambda-restricted immunoglobulin.	Not reported.	Not reported.
**Hsu 2011** [17]	65	M	Progressive lower leg weakness and numbness over 2 years followed by quadriplegia.	MRI T2: C5-C6 hyperintense lesion with C6 vertebral body destruction.	Laminectomy with resection.	Dramatic improvement in muscle strength, at the time of the report the patient would walk without limitation.	Positive Congo red stain with a previous biopsy revealing β2M deposits.	Developed bilateral carpal tunnel syndrome after 8-years of regular hemodialysis.	Not reported.
**Sueyoshi 2011** [18]	71	M	Urinary incontinence, sensory disturbances in the arms, and became unable to ambulate.	MRI: Contrast-enhancing extradural mass at C3-C4 with severe osteolysis of the vertebral body.	C3-C7 laminoplasty, bilateral C5 foraminotomy, and lumbar spinal fusion.	Upper limb weakness moderately improved; other symptoms partially improved and did not worsen.	Positive for amyloid deposits. Detected ATTRwt using mass spectrometric analysis.	No; however, nerve conduction study revealed prolonged sensory latency of the median nerve consistent with CTS.	MRI showed spinal canal stenosis due to osteophytes at L3-L4.
**Takeshima 2012** [19]	51	F	Progressive headaches.	MRI T2: Solid extradural hypointense lesion at C2	Right sided hemilaminectomies at C1-C2, subtotal resection and right C2 nerve root decompression.	Discharged on day 14 postop with no neurological manifestations. MRI completed at 10-months postop revealed no recurrence.	Histological examination revealed amyloid deposits.	Not reported.	Not reported.
**Hayashi 2012** [20]	75	F	Progressive numbness in upper limbs, fine motor disturbances in fingers bilaterally, and gait disturbance.	MRI: Large hypointense circumferentially enhanced mass in the epidural space from C1-C3.	C2-C4 laminectomy and resection of the mass.	Patient gained increased strength and coordination in extremities, decreased numbness, and was fully ambulatory 2-months postop.	Positive Congo red stain with immune-histochemical testing indicating non-AA amyloid.	Not reported.	Not reported.
**Werner 2013** [21]	77	F	Worsening syncope and altered mental status, acute weight loss, gradual weakness in the upper and lower limbs bilaterally.	MRI: non enhancing mass at C1-C2 with erosive bony changes.	C1-C3 decompression with partial resection, followed by fusion from occiput-C5 using iliac bone graft.	At week 6, regained strength bilaterally in all extremities and became ambulatory. At 2-year follow-up reported intact strength, sensation, and ambulation without aids.	Congo red stained tissue featured yellow-green birefringence under polarized light.	Not reported.	Not reported.
**Nitta 2015** [22]	66	M	1 week of reported dizziness, urinary retention, blunted sensation below the chest, and paraplegia 2-days prior.	MRI T1: Hypointense epidural mass at C7.	Laminectomy and tumor resection.	Paraplegia and urinary retention resolved acutely postoperatively with improved ﻿sensation.	Congo red positive tissue featured apple-green birefringence under polarized light. Immunostaining was positive for β2M amyloid.	No evidence of carpal tunnel.	Not reported.
**Smitherman 2015** [23]	46	F	Lower extremity paralysis, lower body hypoesthesia, and worsening bowel/bladder incontinence.	MRI T1: Intradural extramedullary enhancing lesion at C4-T4.	C4-T4 laminectomy, resection, and posterior spinal fusion	Unchanged neurological examination with no progression at 1-year follow-up.	Congo red stained positively and displayed apple green birefringence under polarized light.	Not reported.	Not reported.
**Shinkino 2016** [24]	57	M	Progressive neck pain, quadriplegia, and numbness of limbs.	MRI T2: C1-C2 hypointense mass	C2-C7 laminoplasty.	At 1-month follow-up, patient exhibited markedly improved symptoms compared to preoperative status.	﻿Amyloid fibrils were densely enhanced with direct fast scarlet staining and showed green birefringence under polarized light. Immunohistochemistry demonstrated a positive finding for β2M.	Carpal tunnel surgery completed 9-years prior.	Thickened LF and PLL.
**Dalolio 2017** [25]	63	M	Acute urinary retention, sensory disturbances in all 4 limbs followed by severe tetraparesis.	MRI T2: intradural extramedullary enhancing lesion at C4-C7.	C4-C7 laminectomy and resection. Underwent Revision surgery consisting of laminoatherectomy at C5-C6 and C6-C7 levels and removal of the lesion.	Discharged day 10 with persistent paraplegia, urinary incontinence, and lower limb sensory deficits with slight improvement in preoperative neurologic symptoms.	Amyloid deposits were confirmed on histological examination.	Not reported.	Thickened LF.
**Rezania 2017** [26]	86	M	Progressive weakness, gait deterioration, falls, and urinary incontinence.	﻿MRI T1: Peripherally enhanced extra-axial mass extending from the clivus to C2.	None, biopsy only.	Died due to complications from metastatic cancer one year later.	Mass spectrometry was performed on Congo red-positive areas which detected transthyretin-related (ATTR) amyloidosis.	Yes.	Yes.
**Schneider 2018** [27]	84	F	Progressive ataxia and dysphagia.	MRI: Non-enhancing soft-tissue mass from the retro-clivus to C2 posteriorly.	Endoscopic trans-nasal resection and posterior stabilization via arthrodesis from occiput-C5.	Discharged 2-weeks postop with improved neuro-motor exam. 2-year follow-up revealed complete resolution of the mass.	Positive Congo red stain and apple green birefringence to polarized light. Presumed AA, negative for kappa and lambda AL.	Not reported.	Not reported.
**Rotter 2019** [28]	58	M	Neck pain radiating to left arm, bilateral upper limb weakness over several months.	MRI T2: hypointense, contrast-enhancing mass at C1-C2.	C2 laminectomy and gross total resection.	One-month postop assessment revealed improved strength in upper and lower extremities.	Congo red showed green birefringence under cross-polarized light. Liquid chromatography tandem mass spectrometry detected an AL-kappa amyloid peptide profile.	Not reported.	Not reported.
**Giorgi 2021** [29]	57	F	Progressive loss of upper and lower limb strength and sensitivity in addition to headaches.	MRI: Hypointense solid mass at C1-C2.	C1 laminectomy, followed by occiput-C5 fixation.	Neurological deficits improved immediately after surgery; 1-year follow-up revealed no signs of myelopathy progression.	Positive Congo red reaction and were positive for β2M immunostaining.	Not reported.	Not reported.

*CTS Hx* Carpal Tunnel Syndrome history, *β2M* beta-2-microglobulin, *ATTR* Transthyretin, *MM* Multiple Myeloma, *AL* Amyloid Light Chain, *AA* Serum Amyloid A, *ATTRwt* Transthyretin wild type, *LF* Ligamentum Flavum, *PLL* Posterior Longitudinal Ligament

Previous research has shown that presenting symptoms of cervical amyloidoma most often includes pain, discomfort, subjective weakness, or sensory disturbances. Common physical exam findings include objective weakness, paralysis, and hyperreflexia [21]. MRI imaging often reveals hypointense structures on T1 and T2 images and mostly occupies the epidural space and bone at the C1, C2, C6 and C7 levels [2, 21]. These findings were concurrent with our case and with what has been previously reported in ATTR-specific cases. The differential for C1-C2 pannus is broad and includes primary or metastatic bone tumours, infectious processes, rheumatoid arthritis, calcium pyrophosphate deposition, pigmented villonodular synovitis, gout, osteoarthritis, brown tumour, or amyloidoma [13, 39].

Cervical amyloidoma patients aged ranged from 45 to 86 with an average age of 66 ± 11.9, where 16 patients were male (61.5%) and 10 were female (38.5%). Notably, our patient alongside two other previously reported ATTR cases had a history significant for CTS [19, 25]. As well, the remaining previously published ATTR case report mentioned their patient having prolonged sensory latency of the median nerve [10]. Spinal stenosis due to ligamentous hypertrophy was only reported in the case by Rezania et al. and not described in the remaining ATTR reports [19]. In general, our findings complement previous work and illustrates the potential for CTS and LF hypertrophy to proceed in patients with unknown pannus-like masses compressing the spinal cord, causing neurological deficits on examination.

Medical therapeutics for ATTR have been developed and are classified as transthyretin silencers, stabilizers, and amyloid fibril disruptors/degraders [40]. Such therapeutic agents have been employed to treat cardiac ATTR by targeting various stages of the disease process in hopes of halting progression and possibly reversing aggregation [40]. To date, the mainstay treatment for amyloidoma is surgical excision, with some reports describing the benefits of adjuvant radiotherapy or chemotherapy for patients who were not surgical candidates [2]. A report by Farrell et al. demonstrated improved clinical outcomes for AL cervical amyloidoma using 6 cycles of bortezomib and dexamethasone [8]. Our review revealed no reports describing the use of ATTR-specific therapeutics in the management of spinal amyloidoma. Thus, the real benefits of the treatment of amyloidoma using radiotherapy, chemotherapy, or ATTR-specific therapeutics have not yet been demonstrated or described at all.

Patients diagnosed with localized amyloid deposits have an excellent prognosis due to the lack of systemic involvement and benign tumour-like growth. Interventions most often consist of decompressive surgery with and without fusion depending on the extent of resection [2]. Post-operative outcomes often improve or completely resolve presenting symptoms and are complimented with low reported rates of recurrence and mortality [2, 21]. However, it should be noted that no long-term follow-up reports have been published yet.

In our case, the patient presented with pain, reduced motion, loss of balance, and poor subjective strength which are consistent with common presentations found in previously published literature. Patients with solitary spinal amyloidoma are important to distinguish from other diagnoses due to their favourable outcomes; however, physicians need to maintain clinical suspicion and obtain tissue biopsies to confirm the diagnosis as this is the only proven method of authentication to date. Additionally, CTS and spinal stenosis caused by hypertrophied LF have been attributed to ATTR amyloidosis; hence, patients who develop focal spinal lesions whose history is significant for either or both should maintain clinical suspicion for solitary spinal amyloidoma as these conditions may appear years prior.

## Data Availability

Data sharing is not applicable to this article as no datasets were generated or analyzed during the current study.

## Abbreviations

AA: Serum Amyloid A
Aβ2M: β2 Microglobulin
AL: Immunoglobulin Light Chain
ATTR: Transthyretin amyloidosis
ATTRwt: Transthyretin wild type
ATTRv: Transthyretin variant
CT: Computed Tomography
CTS: Carpal Tunnel Syndrome
LF: Ligamentum of Flavum
MRI: Magnetic Resonance Imaging
NCS: Nerve Conduction Study
